# Pathways to promotion: A road map for growth and impact in academic medicine

**DOI:** 10.1002/jhm.70211

**Published:** 2025-11-04

**Authors:** Elizabeth A. Murphy, Keri Holmes‐Maybank, Alfred Burger, Rebecca Harrison

**Affiliations:** ^1^ Section of Hospital Medicine University of Chicago Medicine Chicago Illinois USA; ^2^ Department of Medicine Medical University of South Carolina Charleston South Carolina USA; ^3^ Department of Medicine Icahn School of Medicine at Mount Sinai New York New York USA; ^4^ Section of Hospital Medicine Oregon Health & Sciences University Portland Oregon USA

## Abstract

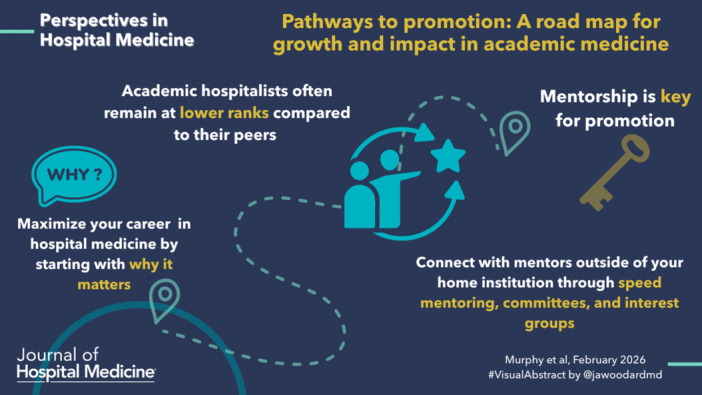

As medical students, we often looked at full professors or rapidly advancing junior faculty members with admiration and curiosity, wondering how they reached their current positions. The truth is, it′s often the promotion process that played a pivotal role in their academic success. Promotion is often well worth pursuing for one′s own professional growth and fulfillment and for the opportunity to contribute to the profession of hospital medicine.

Academic hospitalists often remain at lower ranks compared to their peers in Departments of Medicine or Pediatrics due to multiple barriers. These include a lack of senior mentors in hospital medicine at the associate professor and professor levels, a paucity of scholarly experience, training, and/or skills, an emphasis on peer‐reviewed publications in the promotions process, and disproportionate clinical obligations.^1^, ^2^, ^3^ The relative youth of the field of Hospital Medicine and its origin as a clinical rather than research discipline contributes to the dearth of associate professor and professor ranked hospitalists. This is reflected in their modest scholarly productivity compared with peers in other fields.^4^ The shortage of experienced mentors further hampers development of academic hospitalists, leading to stagnation in career progression.^5^

As the focus on clinical work and performance metrics intensifies, there may be fewer opportunities for academic pursuits. This results in fewer publications and increasingly constrained availability for collaborative academic activities outside one′s institution. Yet, paradoxically, academic expectations for hospitalists are intensifying. This shift is driven by multiple forces, including the continued formalization of hospital medicine as an academic discipline, institutional efforts to align promotion standards across departments, and the expanding role of hospitalists in education, quality improvement, and leadership.^6^, ^7^, ^8^ At academic medical centers, where hospitalists frequently serve as core teaching faculty and clinical leaders, there is increasing institutional pressure to support and evaluate them through traditional academic lenses.^9^ Regardless of this broadening of roles and responsibilities, the barriers to promotion loom large in Hospital Medicine. Unsurprisingly, the newer generation of hospitalists express ambivalence about the benefits of an academic career and the prospect of promotion.^10^ They pose a crucial question: Why should hospitalists pursue an academic path, dedicating time and energy to a mandated promotion pathway that could otherwise be spent on clinical care or one′s personal life?

The answer to this question varies for each academic hospitalist. Most seek promotion for the opportunity to grow professionally, clinically, and personally, gaining improved access to professional opportunities and recognition for their contributions. Achieving higher ranks in academic promotion increases respect from specialists at academic medical centers, enhances credibility in academic and clinical pursuits, including the ability to support or mentor junior faculty within and beyond hospital medicine, and potentially leads to higher compensation.

Academic promotion provides a framework for long‐term growth and development. Prioritizing promotion helps hospitalists focus on their academic passions amidst competing clinical demands, encouraging them to identify and cultivate the meaning in their work. Promotion necessitates the creation of a cohesive body of academic work and the opportunity for feedback about one′s accomplishments, distribution of activities, and goals. To maximize a career in hospital medicine, starting with why it matters—whether driven by a passion for teaching, solving clinical systems problems, or generating new knowledge to inform clinical care delivery—is essential. This reflection involves asking these questions repeatedly, reflecting upon meaning, advocating for oneself within the division or department, and adjusting career trajectories as priorities evolve.

Promotion serves as a roadmap for academic hospitalist success, offering structure through defined requirements and timelines. Pursuing and achieving promotion opens up new opportunities, such as teaching and leadership roles, albeit with variations in expectations among institutions. Regional and national involvement, including leadership roles in professional organizations, collaboration on projects, and co‐authorship on publications, as well as invitations to lecture, enhances recognition and reputation in academic hospital medicine. These efforts propel academic hospitalists toward success beyond checklist accomplishments, provides a broader view of the depth and impact of hospital medicine as a career and encourages forming lifelong professional friendships.

Most academic institutions recognize that mentorship and coaching relationships are critical to faculty success and advancement. Academic hospitalist leaders are uniquely positioned to foster these relationships—both locally and beyond—through regional and national engagement. Participation in networks like the Society of Hospital Medicine′s (SHM) speed mentoring and one‐on‐one mentoring programs and Pediatric Hospital Medicine′s meet the professors and speed mentoring sessions provides access to mentors outside one′s home institution, broadening perspectives and career guidance. Involvement in SHM′s national meeting, committees, and interest groups, as well as SGIM′s Academic Hospitalist Commission, cultivates not only mentorship but also peer coaching and community among hospitalists facing similar challenges. These connections reduce isolation and demystify the promotion process. Moreover, they allow hospitalists to establish thought leadership in the field, reinforce professional identity, and eventually mentor others, advancing both institutional and national academic missions.^11^

Promotion to rank of associate professor and full professor enhances influence locally, regionally, and nationally, bolstering credibility in academics and leadership. This progression creates opportunities to contribute to important clinical, educational, and research missions through participation in influential college, university, department, or hospital committees. Increasing the number of highly ranked academic hospitalists strengthens the legitimacy of hospital medicine and intensifies the power of hospitalist advocacy for local and national healthcare policy. These opportunities deepen professional impact and amplify the meaning derived from academic hospitalist careers.

Importantly, academic faculty with dedicated time to pursue professionally meaningful nonclinical activities are protected against burnout.^12^ Higher academic rank presents hospitalists with more opportunities to focus on their career passions within academic hospital medicine and help move future generations forward. Promotion provides a roadmap to success, expands mentorship opportunities, fosters a professional community, and increases influence institutionally and nationally. Understanding why promotion matters is crucial for developing a fulfilling, long‐term career in academic hospital medicine.

## CONFLICT OF INTEREST STATEMENT

The authors declare no conflicts of interest.

## References

[1] Herzke C , Bertram A , Stein A , Yeh HC , Cofrancesco, Jr. J. Promotion of academic hospitalists: room for improvement. Am J Hosp Med. 2021;5(4):5.

[2] Reid MB , Misky GJ , Harrison RA , Sharpe B , Auerbach A , Glasheen JJ . Mentorship, productivity, and promotion among academic hospitalists. J Gen Intern Med. 2012;27:23‐27. 21953327 10.1007/s11606-011-1892-5 PMC3250536

[3] Leykum LK , Parekh VI , Sharpe B , Boonyasai RT , Centor RM . Tried and true: a survey of successfully promoted academic hospitalists. J Hosp Med. 2011;6(7):411‐415. 21916004 10.1002/jhm.894

[4] Renner CS , Sumarsono A , Mathew A , et al. Scholarly productivity and growth of academic hospital medicine full professors. J Hosp Med. 2022;17(7):509‐516. 35761782 10.1002/jhm.12894

[5] Lin D , Schmidt RM , Shah C , et al. A facilitated peer mentoring program with a dedicated curriculum to foster career advancement of academic hospitalists. MedEdPORTAL. 2023;19:11366. 38076293 10.15766/mep_2374-8265.11366 PMC10704005

[6] Wachter RM , Goldman L . The emerging role of “hospitalists” in the American health care system. N Engl J Med. 1996;335(7):514‐517. 8672160 10.1056/NEJM199608153350713

[7] Bunton SA , Corrice AM , Mallon WT . Predictors of promotion and tenure in academic medicine: a national study of faculty in U.S. medical schools. Acad Med. 2012;87(9):1235‐1241.

[8] Larsen T , Simon W , Lazarus ME , Patel S . The role of the hospitalist in the clinical education of medical students. Brown Hospital Medicine. 2023;2(4). 10.56305/001c.87819. PMC11864450 40028309

[9] Wiese JG , Wachter RM , Flanders SA . The hospitalist movement 20 years later: a summary of the impact on education, research, and practice. J Hosp Med. 2016;11(10):701‐704.27130579

[10] Cumbler E , Rendón P , Yirdaw E , et al. Keys to career success: resources and barriers identified by early career academic hospitalists. J Gen Intern Med. 2018;33:588‐589. 29423628 10.1007/s11606-018-4336-7 PMC5910353

[11] Coates WC . Being a mentor: what's in it for me? Academic Emergency Medicine. 2012;19(1):92‐97. 22221391 10.1111/j.1553-2712.2011.01258.x

[12] Shanafelt TD , West CP , Sloan JA , et al. Career fit and burnout among academic faculty. Archives of Internal Medicine. 2009;169(10):990‐995. 19468093 10.1001/archinternmed.2009.70

